# Modelling study to estimate the health burden of foodborne diseases: cases, general practice consultations and hospitalisations in the UK, 2009

**DOI:** 10.1136/bmjopen-2016-011119

**Published:** 2016-09-13

**Authors:** Sarah J O'Brien, Tricia L Larose, Goutam K Adak, Meirion R Evans, Clarence C Tam

**Affiliations:** 1 University of Liverpool Institute of Infection and Global Health, Liverpool, UK; 2 NIHR Health Protection Research Unit in Gastrointestinal Infections, The Farr Institute@HeRC, University of Liverpool, Liverpool, UK; 3 Department of Infectious Disease Epidemiology, London School of Hygiene and Tropical Medicine, London, UK; 4 Department of Public Health and General Practice, Norwegian University of Science and Technology, Trondheim, Norway; 5 Department of Gastrointestinal, Emerging & Zoonotic Infections, Public Health England Centre for Infectious Disease Surveillance and Control, London, UK; 6 Institute of Primary Care and Public Health, Cardiff University, Cardiff, UK; 7 Saw Swee Hock School of Public Health, National University of Singapore, Singapore, Singapore

**Keywords:** Foodborne disease, *Salmonella*, *Campylobacter*, Norovirus, Food safety, *Clostridium perfringens*

## Abstract

**Objective:**

To generate estimates of the burden of UK-acquired foodborne disease accounting for uncertainty.

**Design:**

A modelling study combining data from national public health surveillance systems for laboratory-confirmed infectious intestinal disease (IID) and outbreaks of foodborne disease and 2 prospective, population-based studies of IID in the community. The underlying data sets covered the time period 1993–2008. We used Monte Carlo simulation and a Bayesian approach, using a systematic review to generate Bayesian priors. We calculated point estimates with 95% credible intervals (CrI).

**Setting:**

UK, 2009.

**Outcome measures:**

Pathogen-specific estimates of the number of cases, general practice (GP) consultations and hospitalisations for foodborne disease in the UK in 2009.

**Results:**

Bayesian approaches gave slightly more conservative estimates of overall health burden (∼511 000 cases vs 566 000 cases). *Campylobacter* is the most common foodborne pathogen, causing 280 400 (95% CrI 182 503–435 693) food-related cases and 38 860 (95% CrI 27 160–55 610) GP consultations annually. Despite this, there are only around 562 (95% CrI 189–1330) food-related hospital admissions due to *Campylobacter*, reflecting relatively low disease severity. *Salmonella* causes the largest number of hospitalisations, an estimated 2490 admissions (95% CrI 607–9631), closely followed by *Escherichia coli* O157 with 2233 admissions (95% CrI 170–32 159). Other common causes of foodborne disease include *Clostridium perfringens*, with an estimated 79 570 cases annually (95% CrI 30 700–211 298) and norovirus with 74 100 cases (95% CrI 61 150–89 660). Other viruses and protozoa ranked much lower as causes of foodborne disease.

**Conclusions:**

The 3 models yielded similar estimates of the burden of foodborne illness in the UK and show that continued reductions in *Campylobacter*, *Salmonella*, *E. coli* O157, *C. perfringens* and norovirus are needed to mitigate the impact of foodborne disease.

**Strengths and limitations of this study**

- This is the first burden of foodborne illness modelling study to incorporate empirical data and prior information from a systematic review together with Bayesian methodology for estimating the proportion of infectious intestinal disease that is transmitted through contaminated food.
- Our estimates are based on high-quality data sets, including directly observed, pathogen-specific incidence data.
- Our methods take full account of parameter uncertainties.
- There are several data gaps which need to be filled including pathogen-specific mortality estimates, and information on morbidity in vulnerable populations such as immunocompromised people, older people and pregnant women.

## Introduction

Food safety is a global priority.^1^ To have maximum impact, the design and funding of food safety interventions need to take account the overall burden of foodborne disease and the contribution made by each pathogen. Developing better methods for estimating the true burden of foodborne disease has been the focus of international efforts for over a decade.^1–8^ This is problematic for various reasons: people usually present with non-specific symptoms of infectious intestinal disease (IID), only a fraction of cases are confirmed by laboratory testing, and not all are reported to national public health surveillance. IID then needs to be attributed to transmission route (foodborne, waterborne, animal-to-person, person-to-person or environment-to-person), which can be difficult if robust epidemiological information is lacking. In a recent population-based, prospective study in the UK (known as the IID2 study) we found that IID affected around one in four people each year (∼17 million cases in 2009).^9^ We used novel methods to estimate, for each pathogen, the proportion of IID attributable to food and the health burden of UK-acquired foodborne disease.

## Methods

### Data sources

#### The IID studies

Two population-based studies of IID have taken place in the UK (box 1). The first (IID1 study) was conducted in England in 1993–1996,^9^ and the second (IID2 study) took place across the whole of the UK in 2008–2009.^10–11^ Both comprised (1) a prospective cohort study of people living in the community and (2) a prospective study of patients presenting to general practice (GP) with symptoms of IID. Samples were obtained for laboratory testing from symptomatic cases in the cohort and from patients presenting to GP and tested using comprehensive microbiology algorithms.^11^ ^12^ The case definitions were identical in both studies, and incidence rates of IID in the community and GP consultation rates for IID were calculated. Data on healthcare use, captured by questionnaires, gave estimates of hospitalisation rates.

Box 1 **Sample sizes in the IID1 and IID2 studies^9^ ^10^**

IID1, England, August 1993—January 1996:

 Prospective Cohort Study: N=9776;

 General Practice Presentation Study: N=4026.

IID2, UK, April 2008—August 2009:

 Prospective Cohort Study: N=7033;

 General Practice Presentation Study: N=991.

#### Outbreak surveillance data

The four UK national surveillance centres provided data on general outbreaks of IID occurring between 1 January 2001 and 31 December 2008 (n=2965; table 1). There were substantial changes to outbreak reporting in 2009. Prior to 2009 Public Health England (PHE) collected data on all gastrointestinal infection outbreaks no matter what the transmission route was, that is, foodborne, waterborne, person to person, environment to person and animal to person. In 2009 PHE limited the collection of outbreak data on ‘non-foodborne outbreaks’ to ‘gastrointestinal outbreaks including illnesses associated with recreational water exposure, environmental exposure at outdoor events example contact with mud, contact with animals or their faeces and outbreaks of verocytotoxin-producing *Escherichia coli* (VTEC) mediated through person-to-person transmission’. Thus the data collected from December 2008 represented a subset of outbreaks rather than all outbreaks. This affected the proportion of illnesses assessed as foodborne because the denominators of outbreaks and cases in outbreaks changed substantially as a result of changes in reporting definitions. This was particularly problematic for pathogens such as norovirus and *Cryptosporidium*.

**Table 1:** Summary of outbreak data for food attribution by pathogen, UK 2001–2008

	Foodborne outbreaks			Cases in foodborne outbreaks			
Organism	Foodborne	All outbreaks	Per cent	Cases	All cases	Per cent	Source
Bacteria							
*C. perfringens*	45	60	75.0	1691	1964	86.1	Outbreak surveillance
*Campylobacter*	31	44	70.5	373	761	49.0	Outbreak surveillance
*E. coli O157*	25	86	29.1	564	1041	54.2	Outbreak surveillance
*Listeria*	2	2	100.0	6	6	100.0	Outbreak surveillance
*Salmonella*	266	308	86.4	7128	7892	90.3	Outbreak surveillance
*Shigella*	4	11	36.4	65	310	21.0	Outbreak surveillance
Protozoa							
*Cryptosporidium*	4	65	6.2	415	1375	30.2	Outbreak surveillance
*Giardia*	1	7	14.3	106	159	66.7	Outbreak surveillance
Viruses							
Adenovirus	–	–	–	–	–	–	No outbreaks reported
Astrovirus	0	18	0.0	0	283	0.0	Outbreak surveillance
Norovirus	61	2228	2.7	1500	58 855	2.5	Outbreak surveillance
Sapovirus	–	–	–	–	–	–	No outbreaks reported
Rotavirus	1	136	0.7	30	2338	1.3	Outbreak surveillance

*C. perfringens*, *Clostridium perfringens*; *E. coli*, *Escherichia coli.*

For each outbreak, information was available on the following: outbreak setting, number of cases affected, number of cases hospitalised, main mode(s) of transmission, pathogen identified and, for outbreaks involving contaminated foods, the implicated food vehicle (where this was ascertained). For this study, point source or disseminated outbreaks involving contaminated food, and outbreaks involving contaminated food with subsequent person-to-person transmission, were considered to be foodborne. In total, there were 446 outbreaks involving foodborne transmission that were available for analysis.

#### Systematic literature review

We conducted a systematic literature review according to the Preferred Reporting Items for Systematic Reviews and Meta-Analyses (PRISMA) guidelines.^13^ We searched four databases (MEDLINE, EMBASE, Web of Science and FoodBase—the UK Food Standards Agency's research projects database). The full methodology for our systematic review, and a summary of the results have been reported previously.^14^ We also compared the list of articles that we identified through the systematic review with a list of case–control studies included in a separate, independently published review of case–control study methods for enteric infection.^15^ We identified 32 articles published between 1 January 2001 and 31 December 2011 with relevant information that allowed us to determine the percentage of cases of IID attributable to foodborne transmission (see also online supplementary technical appendix). The Bayesian priors were based on uniform distributions, which essentially assumed that any value within a specified range is likely equal. The lower and upper bounds of the distribution were determined by the lowest and highest estimates from studies found in the literature review. So for example, the reported range for foodborne *Campylobacter* was between 42% and 80%, and these percentages formed the lower and upper bounds used for the uniform prior (see online supplementary technical appendix).

10.1136/bmjopen-2016-011119.supp1 Supplementary technical appendix

### Modelling approach

We developed a model to estimate the number of cases, GP consultations and hospital admissions of UK-acquired foodborne disease due to 13 major enteric pathogens: *Clostridium perfringens, Campylobacter, E. coli O157*, *Listeria*, *Salmonella* (non-typhoidal), *Shigella*, *Cryptosporidium*, *Giardia*, adenovirus, astrovirus, norovirus, rotavirus and sapovirus. The basic model was:
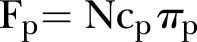

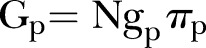

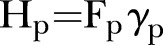
where F_p_, G_p_ and H_p_ represent, respectively, the estimated number of UK-acquired foodborne disease cases, GP consultations or hospital admissions for pathogen p in 2009. C_p_ is the UK incidence of IID due to pathogen p and g_p_ is the GP consultation rate for IID due to pathogen p. The constant, N, is the mid-2009 population of the UK. The two parameters, π_p_ and γ_p_, represent, respectively, the proportion of IID cases due to pathogen p that are transmitted through food, and the proportion of cases due to pathogen p that are hospitalised. We assumed that foodborne cases were equally likely to consult a GP or be hospitalised as non-foodborne cases.

We used various data sources to inform model parameters. The data available for each pathogen are summarised in the online supplementary technical appendix tables A1–A3. We used two modelling approaches: a Monte Carlo simulation approach and a Bayesian approach. In the Monte Carlo approach, the parameters π_p_ and γ_p_ were defined by β distributions fitted to empirical bootstrap samples of UK outbreak data; in the Bayesian approach, these parameters were modelled as binomial quantities and given priors informed by published studies and hospitalisation data from previous studies in the UK. Model details are given in the online supplementary technical appendix.

### Pathogen-specific rates of IID (c_p,_ γ_p_)

We obtained data from the IID2 study on population incidence and GP consultation rates for IID, and their associated uncertainty, for the above pathogens.^10^ For *Shigella*, no cases were found in IID2 so we applied the reporting ratio from IID1 (the ratio of community cases to laboratory-confirmed cases reported to national surveillance) to the number of cases reported in 2009 and divided this by the mid-2009 UK population to obtain the overall shigellosis rate.^11^ Similarly, we estimated GP consultation rates by applying the reporting ratio from IID1 (the ratio of GP consultations to laboratory-confirmed cases reported to national surveillance) to the number of laboratory reports in 2009. We accounted for uncertainty in incidence estimates by sampling 100 000 times from the distribution of reporting ratios estimated in IID1. For *Listeria*, no incidence data were available from IID1 or IID2 so we used the number of laboratory reports for listeriosis in 2009 as a conservative population incidence estimate.

### Proportion of cases transmitted through food (π_p_)

#### Estimating the proportion of cases transmitted through food

We used data on outbreaks reported to national surveillance systems between January 2001 and December 2008 to estimate the proportion of cases transmitted through food. For each pathogen, we computed empirical estimates for π_p_ by obtaining 4999 bootstrap samples of the proportion of cases in outbreaks that resulted from foodborne transmission. We then fitted a β function to the resulting distribution using maximum likelihood. For *Cryptosporidium* and *Giardia*, this approach gave an unrealistically high estimate for the proportion of cases transmitted through food because, of the few outbreaks that were reported, those involving foodborne transmission were larger. For these two pathogens, we used instead the proportion of outbreaks that were foodborne as an estimate of π_p_, as was done in a previous study.^5^ For adenovirus and sapovirus, for which no outbreaks were reported, we used parameters derived from analysis of rotavirus and norovirus outbreaks respectively. For pathogens for which all outbreaks or no outbreaks were foodborne, we specified limits to the fitted β distributions as described in the online supplementary technical appendix. The a and b parameters from the fitted β distributions were then used in the Monte Carlo simulations (see Model 1 below).

### Prior distributions for the proportion of cases transmitted through food (π_p_)

We obtained prior distributions for the π_p_ parameters from the systematic literature review. We divided the retrieved articles into two categories: food attribution studies (Group A) and others (Group B). In Group A studies the proportion of cases transmitted through food was estimated for several pathogens, through expert elicitation or retrospective data reviews. Group B were primarily pathogen-specific case–control studies or studies using microbiological typing for source attribution. For Group A and Group B studies, we defined uniform distributions for π_p_, based on the minimum and maximum estimates of the proportion of cases transmitted through food in these studies, for pathogens with at least two published studies. Where the observed proportion from outbreak data fell outside the limits of this uniform distribution, we arbitrarily allowed the lower or upper limit of the distribution to extend by 0.1 beyond the observed value.

### Pathogen-specific hospitalisation (γ_p_)

Data on hospitalisations were available only for outbreaks reported in England and Wales. For each reported outbreak, excluding those in hospitals or residential institutions, we computed the proportion of cases hospitalised by causative organism. We based hospitalisation estimates on all outbreaks with the available data, as we found no major differences in hospitalisation between foodborne and other outbreaks. To account for uncertainty in these parameters, we fitted β distributions to bootstrapped data as detailed above for π_p_, but additionally weighting by outbreak size (see online supplementary technical appendix). For adenovirus and sapovirus, we used parameters derived from analysis of rotavirus and norovirus outbreaks, respectively. Bootstrap estimates with fitted β distributions by pathogen are shown in the online supplementary technical appendix.

### Prior distributions for pathogen-specific hospitalisation (γ_p_)

We used pathogen specific, β-distributed priors for γ_p_. The β parameters were informed by an analysis of hospitalisation data from the IID1 and IID2 studies (see online supplementary figure A1 and technical appendix).

Estimating food-related IID cases, GP consultations and hospitalisations (F_p_, G_p_, H_p_). We obtained estimates of the number of foodborne cases, GP consultations and hospitalisations using three different approaches. In Model 1, we used the Monte Carlo simulation to draw values at random from each parameter distribution. In Model 2, we used a Bayesian approach that included parameters for the prior distributions of γ_p_ from the IID1 and IID2 studies and for π_p_ from Group A studies as described above. These priors were used, together with the outbreak data, to obtain posterior distributions for these parameters, which were then used in the model. This model could not be applied to sapovirus, because none of the identified studies had information about this pathogen. Model 3 had the same structure as Model 2, except that Bayesian priors for π_p_ from Group B studies were used instead. This model was applied to *Campylobacter*, *E. coli O157*, *Listeria* and *Salmonella*, for which sufficient data from published studies were available. A full description of model parameters is given in the online supplementary technical appendix.

For each model, we carried out 100 000 simulations, discarding the first 10% and retaining the model outputs for every 10th simulation. We checked model convergence graphically by plotting parameter values over time to verify adequate mixing, plotting autocorrelograms and comparing density plots for outcome variables by tertile of the simulation chain. We summarised model outputs using the median and central 95% of the posterior distributions to obtain point estimates and 95% credible intervals (CrI) for the number of food-related cases, GP consultations and hospitalisations by pathogen. We conducted the analyses using Stata V.12.1, WinBUGS and Microsoft Excel software. We used the winbugsfromstata module in Stata to carry out the simulations.^16^

### Ethical considerations

An Ethics Committee favourable opinion was not required. These were secondary analyses of previously collected, publicly available data. All data sets used were completely anonymous and there was no risk of disclosure of personal data.

## Results

### Proportion of cases attributable to foodborne transmission

**Figure 1:**
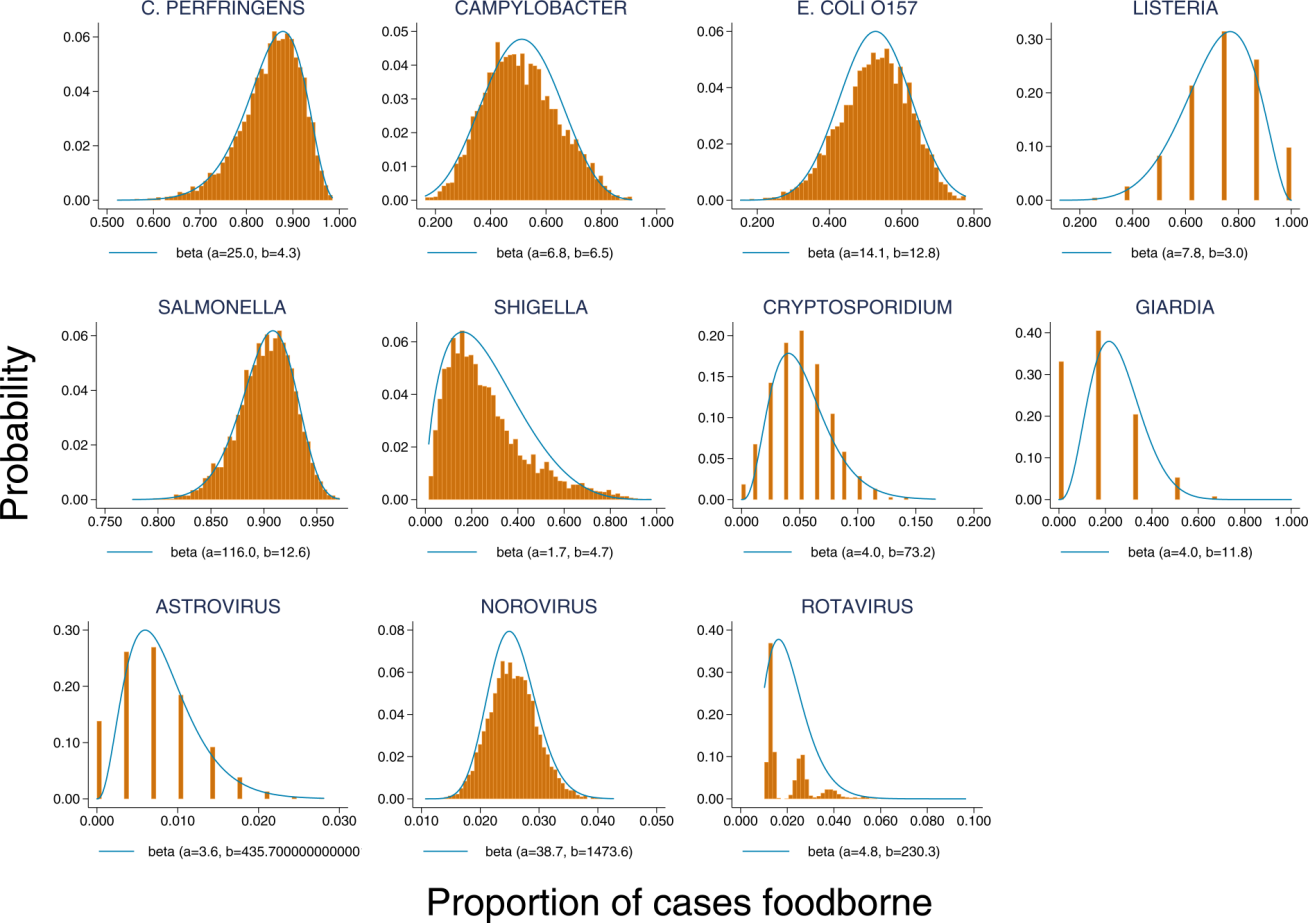
Empirical bootstrap distributions for the estimated proportion of cases due to foodborne transmission based on outbreak data.

Table 1 summarises the outbreak data used for estimating the proportion of cases due to foodborne transmission from outbreak data. The identified studies used to inform Bayesian uniform priors are summarised in the online supplementary technical appendix tables A2 and A3. Figure 1 shows the empirical bootstrap distributions for the estimated proportion of cases due to foodborne transmission based on outbreak data. For most pathogens, the β distribution provided a reasonable fit to the bootstrapped distribution, with the exception of *Giardia*, for which data were sparse, and rotavirus, for which the estimated proportion foodborne transmission was very small. *Salmonella* and *C. perfringens* had the largest estimated proportion of cases attributable to foodborne transmission, each ∼90%. Around 50% of *Campylobacter* and *E. coli O157* cases were estimated to result from foodborne transmission, although there was considerable uncertainty in these estimates as evidenced by the long tails in these distributions. Foodborne transmission accounted for <5% of norovirus cases, while ∼65% of *Giardia* cases, 30% of *Cryptosporidium* cases and 20% of *Shigella* cases were food related.

### Proportion of cases hospitalised

**Figure 2:**
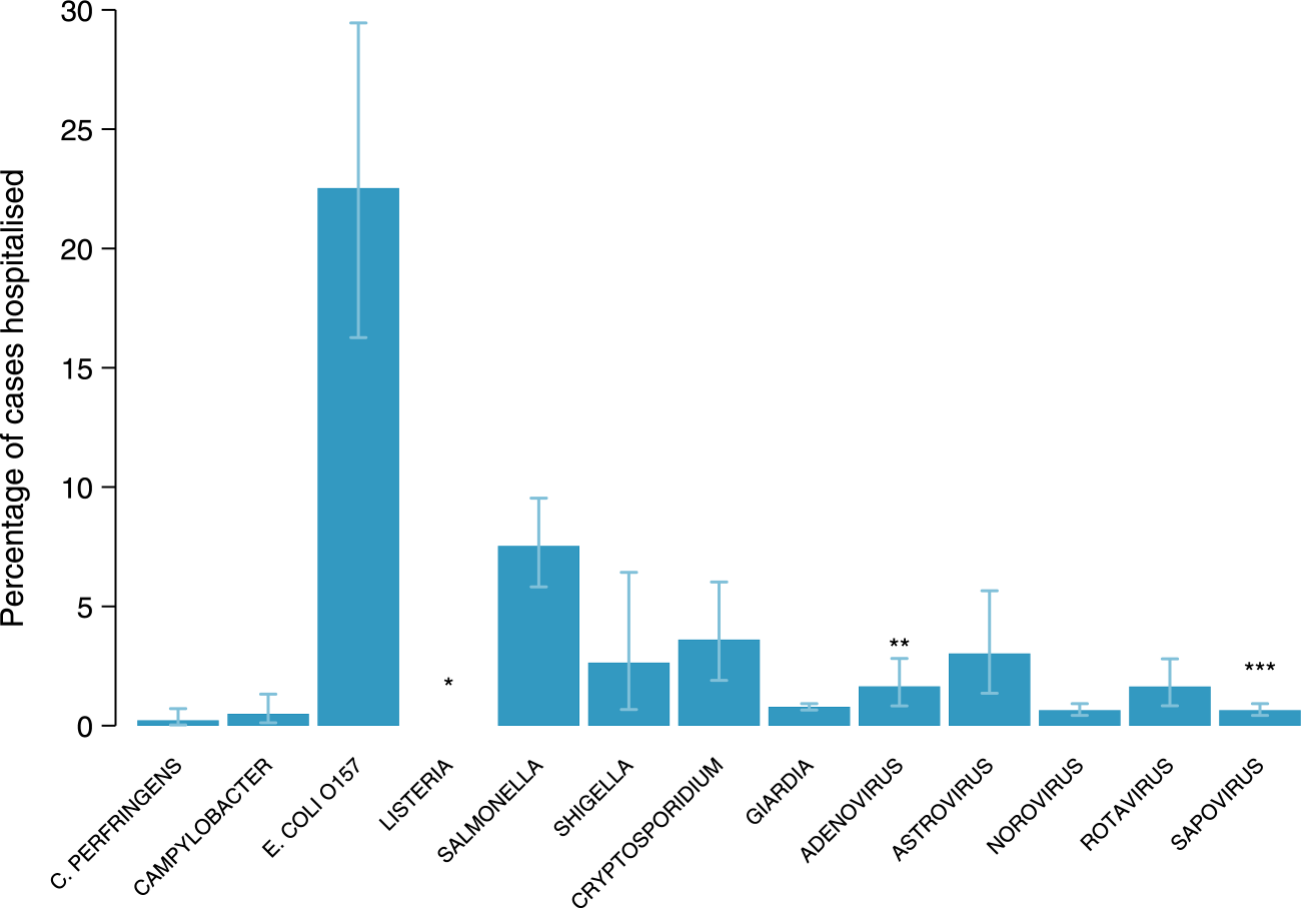
Estimated hospitalisation proportions in reported outbreaks by pathogen, based on the medians of β distributions fitted to outbreak data.

Table 2 summarises the data sources used to inform hospitalisation parameters. Figure 2 shows the estimated hospitalisation proportions in reported outbreaks by pathogen, based on the medians of β distributions fitted to outbreak data. Hospitalisation was particularly high for *E. coli O157* (23%). In contrast, <2% of cases due to *C. perfringens*, *Campylobacter*, *Giardia*, norovirus and rotavirus were hospitalised.

**Table 2:** Summary of hospitalisation data by pathogen, UK 1993–2008

Organism	Hospitalisation in outbreaks					Hospitalisation in IID1 and IID2 studies			
	Hospitalised	Affected	Per cent	Outbreaks with data	Source	Hospitalised	Affected	Per cent*	Source
Bacteria									
*C. perfringens*	2	1120	0.2	21	Outbreak surveillance	2	78	2.6	IID1 and IID2
*Campylobacter*	2	424	0.5	29	Outbreak surveillance	5	441	1.1	IID1 and IID2
*E. coli O157*	197	877	22.5	70	Outbreak surveillance	0	2	33.3	IID1 and IID2
*Listeria*	–	–	–	–	All outbreaks occurred in hospitals	–	–	–	No cases identified
*Salmonella*	419	5527	7.6	217	Outbreak surveillance	4	114	3.5	IID1 and IID2
*Shigella*	4	153	2.6	8	Outbreak surveillance	0	11	8.3	IID1
Protozoa									
*Cryptosporidium*	31	836	3.7	46	Outbreak surveillance	0	50	2.0	IID1 and IID2
*Giardia*	1	137	0.7	5	Outbreak surveillance	1	34	2.9%	IID1 and IID2
Viruses									
Adenovirus	–	–	–	–	No outbreaks reported	0	79	1.3	IID1 and IID2
Astrovirus	2	88	2.3	7	Outbreak surveillance	1	67	1.5	IID1 and IID2
Norovirus	80	12 333	0.6	342	Outbreak surveillance	2	201	1.0	IID1 and IID2
Sapovirus	–	–	–	–	No outbreaks reported	0	77	1.3	IID2
Rotavirus	20	1211	1.7	59	Outbreak surveillance	1	64	1.6	IID2

*Where no hospitalisations were observed, the hospitalised percentage was calculated assuming the next case observed would have been hospitalised (see online supplementary technical appendix).

*C. perfringen*, *Clostridium perfringens*; *E. coli*, *Escherichia coli*; IID, infectious intestinal disease.

### Cases, GP consultations and hospital admissions attributable to foodborne transmission (Model 1)

Table 3 presents estimates of food-related cases, GP consultations and hospital admissions in 2009 from 100 000 Monte Carlo simulations. *Campylobacter* was the most common foodborne pathogen, accounting for 286 000 food-related cases (95% CrI 131 105–532 400) and 39 750 GP consultations (95% CrI 18 890–69 540), but ranked third as a cause of food-related hospital admissions (1376 admissions) behind *Salmonella* (2536 admissions) and *E. coli* O157 (2141 admissions). Foodborne norovirus accounted for 3240 GP consultations (95% CrI 1985–5162), but fewer than 500 hospital admissions. Similarly, other pathogens such as *C. perfringens* and a number of the viruses, while contributing large numbers of cases and GP consultations, were responsible for a modest number of food-related hospital admissions. It should be noted, however, that there was a large degree of uncertainty around these estimates, as demonstrated by the wide 95% CrI.

**Table 3:** Estimates of food-related cases, GP consultations and hospitalisations by pathogen, UK 2009 (Model 1)

Organism	Cases	(95% CrI)	GP consultations	(95% CrI)	Hospital admissions	(95% CrI)
Bacteria						
*C. perfringens*	79 165	(29 310–208 688)	12 610	(5707–27 890)	165	(20–843)
*Campylobacter*	286 000	(131 105–532 400)	39 750	(18 890–69 540)	1376	(289–4607)
*E. coli* O157	9536	(644–146 495)	324	(36–2973)	2141	(143–33 237)
*Listeria*	169	(100–215)	169	(100–215)	–	–
*Salmonella*	33 640	(8286–135 798)	10 030	(4019–24 299)	2536	(608–10 400)
*Shigella*	1274	(90–11 990)	684	(84–2145)	32	(2–378)
Protozoa						
*Cryptosporidium*	2035	(354–10 129)	588	(140–2010)	72	(12–395)
*Giardia*	11 250	(2239–52 878)	1322	(286–4960)	88	(17–415)
Viruses						
Adenovirus	11 920	(3706–28 909)	987	(293–2536)	191	(51–559)
Astrovirus	2362	(594–7180)	180	(41–576)	70	(15–262)
Norovirus	73 420	(50 320–104 000)	3240	(1985–5162)	470	(270–779)
Rotavirus	14 850	(4698–35 330)	1603	(494–3856)	237	(64–688)
Sapovirus	40 770	(26 661–60 230)	2457	(1496–3947)	261	(145–445)
Total	566 391		73 944		7639	

*C. perfringens*, *Clostridium perfringens*; *E. coli*, *Escherichia coli*; GP, general practice.

### Cases, GP consultations and hospital admissions attributable to foodborne transmission (Models 2 and 3)

**Figure 3:**
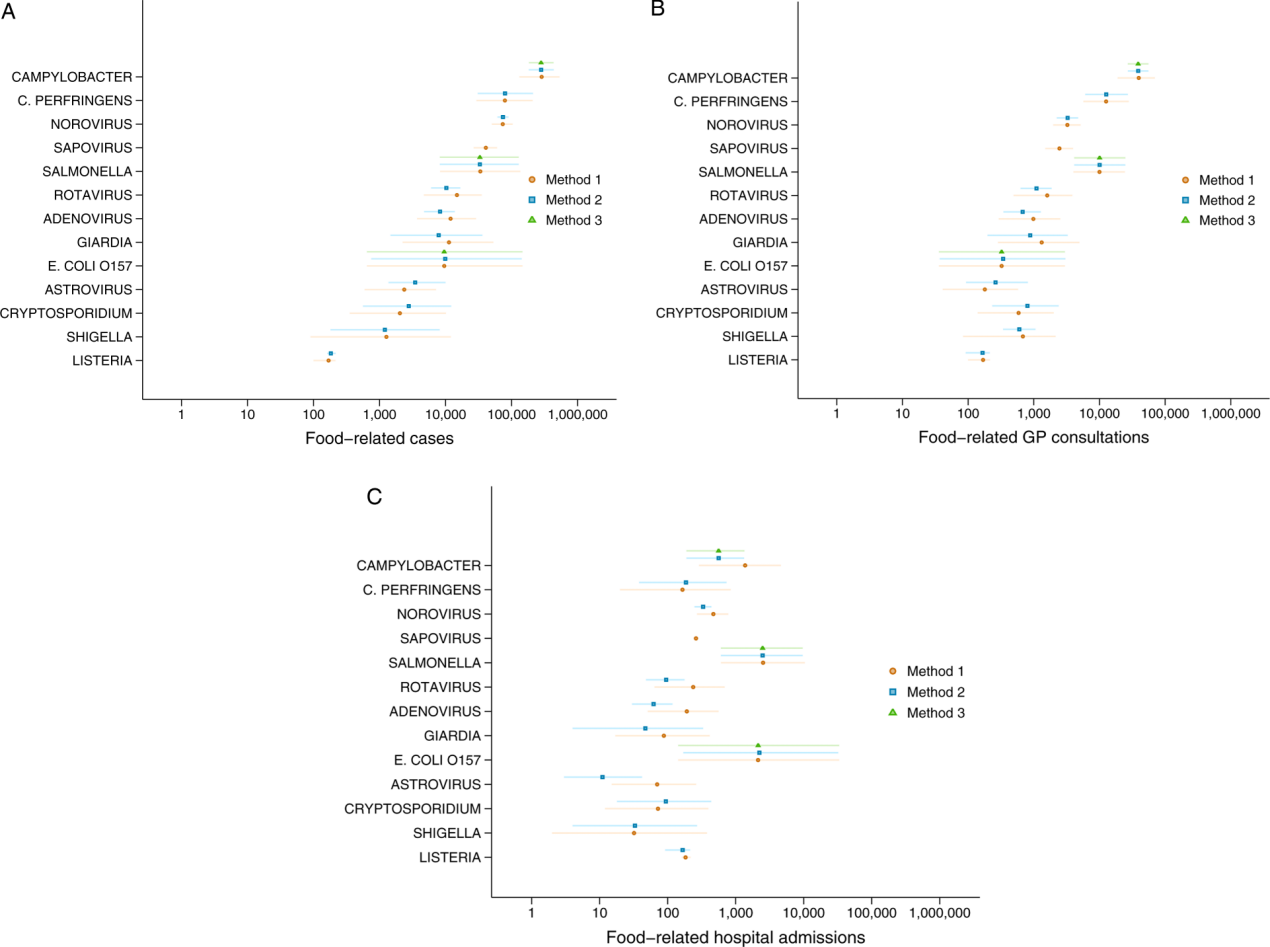
(A) Comparison of estimates from Monte Carlo and Bayesian approaches—food-related cases, UK 2009 (Model 1: Monte Carlo simulation approach; Model 2: Bayesian approach using data from published food attribution studies; Model 3: Bayesian approach using data from published pathogen-specific studies (error bars show 95% CrI). (B) Comparison of estimates from Monte Carlo and Bayesian approaches—food-related general practice consultations, UK 2009 (Model 1: Monte Carlo simulation approach; Model 2: Bayesian approach using data from published food attribution studies; Model 3: Bayesian approach using data from published pathogen-specific studies (error bars show 95% CrI)). (C) Comparison of estimates from Monte Carlo and Bayesian approaches—food-related hospital admissions, UK 2009 (Model 1: Monte Carlo simulation approach; Model 2: Bayesian approach using data from published food attribution studies; Model 3: Bayesian approach using data from published pathogen-specific studies (error bars show 95% CrI)). *C. perfringens*, *Clostridium perfringens*, *E. coli*, *Escherichia coli*.

Estimates of food-related cases, GP consultations and hospital admissions based on the Bayesian approach used in Model 2 are presented in table 4. *Campylobacter* was the most common foodborne pathogen, causing 280 400 (95% CrI 182 503–435 693) food-related cases and 38 860 (95% CrI 27 160–55 610) GP consultations annually. Despite this, there were only 562 (95% CrI 189–1330) *Campylobacter*-related hospital admissions. *Salmonella* caused the largest number of hospitalisations, an estimated 2490 admissions (95% CrI 607–9631), closely followed by *E. coli* O157 with 2233 admissions (95% CrI 170–32 159). Other common causes of foodborne disease included *C. perfringens*, with an estimated 79 570 cases annually (95% CrI 30 700–211 298), and norovirus with 74 100 cases (95% CrI 61 150–89 660). For Model 2, there were insufficient data from the studies we identified to enable estimation of foodborne sapovirus. For *Campylobacter*, *E. coli O157*, *Listeria* and *Salmonella*, further estimates from Model 3 are presented in table 5. The estimates from the three different models are compared in figure 3A–C.

**Table 4:** Estimates of food-related cases, GP consultations and hospitalisations by pathogen, UK 2009 (Model 2)

Organism	Cases	(95% CrI)	GP consultations	(95% CrI)	Hospital admissions	(95% CrI)
Bacteria						
*C. perfringens*	79 570	(30 700–211 298)	12 680	(6072–27 040)	186	(38–732)
*Campylobacter*	280 400	(182 503–435 693)	38 860	(27 160–55 610)	562	(189–1330)
*E. coli* O157	9886	(748–142 198)	342	(37–3030)	2233	(170–32 159)
*Listeria*	183	(161–217)	183	(161–217)	–	–
*Salmonella*	33 130	(8178–128 195)	10 060	(4137–24 710)	2490	(607–9631)
*Shigella*	1204	(181–8142)	602	(341–1060)	33	(4–270)
Protozoa						
*Cryptosporidium*	2773	(562–12 200)	800	(233–2386)	94	(18–436)
*Giardia*	7877	(1467–36 059)	883	(197–3288)	47	(4–332)
Viruses						
Adenovirus	8253	(4734–13 780)	677	(345–1278)	62	(30–118)
Astrovirus	3470	(1368–9991)	262	(93–812)	11	(3–42)
Norovirus	74 100	(61 150–89 660)	3276	(2240–4729)	332	(248–440)
Rotavirus	10 295	(6049–16 730)	1102	(629–1870)	95	(48–177)
Sapovirus*	–	–	–	–	–	–
TOTAL	511 141		69 727		6145	

*For sapovirus, no data were identified in the literature review on the proportion of cases attributable to food, so this model could not be applied.

*C. perfringens*, *Clostridium perfringens*; *E. coli*, *Escherichia coli*; GP, general practice.

**Table 5:** Estimates of food-related cases, GP consultations and hospitalisations by pathogen, UK 2009 (Model 3)

Organism	Cases	(95% CrI)	GP consultations	(95% CrI)	Hospital admissions	(95% CrI)
*Campylobacter*	279 900	(183 100–433 098)	38 820	(27 010–55 580)	561	(189–1343)
*E. coli* O157	9536	(644–146 495)	324	(36–2973)	2141	(143–33 237)
*Listeria*	166	(92–214)	166	(92–214)	–*	–
*Salmonella*	33 130	(8178–128 195)	10 060	(4137–24 710)	2490	(607–9631)
TOTAL	322 732		49 370		5192	

*For Listeria, the number of hospital admissions could not be calculated, as all reported outbreaks occurred in hospitals.

*E. coli*, *Escherichia coli*; GP, general practice.

### Comparing the models

In general, the results from all three approaches were similar for food-related cases and GP consultations. For most organisms, the Bayesian estimates from Model 2 benefited from greater precision. There were differences in the number of food-related hospital admissions estimated by the Monte Carlo and Bayesian approaches for some organisms, notably *Campylobacter*, rotavirus, adenovirus and astrovirus. The differences reflect discordance between outbreak data and data from the IID studies in terms of the hospitalisation rate for these organisms. Where differences were observed, the Bayesian approach gave more conservative estimates of the number of food-related hospital admissions.

For the four pathogens with sufficient data from the literature review to generate estimates from Model 3 (*Campylobacter*, *E. coli O157*, *Listeria* and *Salmonella*), estimates were similar to those from Model 2; however, *Listeria* estimates carried greater uncertainty, because of wide disagreement between the two identified studies regarding the proportion of listeriosis attributable to foodborne transmission. It was impossible to calculate listeriosis hospitalisations because all reported *Listeria* outbreaks occurred in hospitals.

## Discussion

To the best of our knowledge this is the first study to incorporate empirical data and prior information from a systematic review using Bayesian methodology for estimating the proportion of IID that is transmitted through contaminated food. *Campylobacter* is the most common foodborne pathogen in the UK, causing between 182 503 and 435 693 food-related cases and between 27 160 and 55 610 GP consultations annually (based on Model 2 results). Despite this, the number of *Campylobacter*-related hospital admissions is comparatively small, reflecting a generally lower level of acute disease severity compared with other pathogens. In contrast, *Salmonella* and *E. coli* O157 cause the largest number of hospitalisations, an estimated 2490 and 2233 admissions respectively (Model 2), although uncertainty around these estimates is high. Other common causes of foodborne illness include *C. perfringens*, responsible for nearly 80 000 cases annually and norovirus, responsible for nearly 75 000 cases. Other viral agents rank lower as causes of foodborne illness.

Our analysis updates previous estimates for England and Wales in 2000 and expands on them by accounting for uncertainty.^5^ Owing to substantial differences in the analyses, the two sets of estimates are not directly comparable. Other studies investigating the burden of foodborne illness caused by a wide range of pathogens have been carried out in Australia, the USA and the Netherlands.^1^ ^2^ ^6^ ^17^ In the US and Australian studies norovirus was one of the commonest causes of foodborne disease. In the US study, it was also the second most common cause of food-related hospital admissions. Approximately one-quarter of norovirus IID cases in those two studies were attributed to foodborne transmission, whereas our estimate for the UK is <5%. A likely reason for this discrepancy is the definitions of outbreaks that are incorporated in the various modelling studies. Some data sets contain only outbreaks transmitted through food while others, like ours (until 2009), contained all outbreaks of IID no matter what the route of transmission. This means that the proportion of norovirus cases transmitted through food is likely to be overestimated in data sets that contain only outbreaks transmitted through food.

A major strength of our analysis is the availability of directly observed, pathogen-specific incidence data from the recent IID2 study in the UK,^10^ which precludes the need to adjust for underascertainment and requires fewer assumptions about healthcare usage. The use of methods to account fully for parameter uncertainties is an additional strength, and is useful for highlighting areas where data are sparse. This is particularly true for hospitalisation estimates, for which there is a dearth of reliable data. We investigated other sources of hospitalisation data, such as electronic records of inpatient admissions. However, these data lack specific diagnostic codes for certain key pathogens, including *E. coli* O157, and a large fraction of admissions are classified under non-specific diagnostic codes. We therefore used outbreak data to estimate hospitalisation. A potential limitation is that severe cases requiring hospitalisation might be more reliably recorded in outbreak reports, whereas milder cases might be missed. There might genuinely be higher hospitalisation rates in outbreaks than sporadic cases because of higher dose exposures or different populations might be affected in outbreaks. Alternatively, outbreaks with more hospitalised cases might be more likely to be investigated and reported. This would tend to overestimate hospitalisation rates. Such a bias is possible in the *E. coli* O157 data, where estimates for hospitalisations were considerably higher than for GP consultations. Alternatively, the severity of this disease could mean that cases are admitted directly to hospital without first consulting a GP. Our Bayesian models additionally incorporated prior information on hospitalisation rates from IID1 and IID2. For most pathogens, the two types of models gave similar results. However, the number of hospitalisations in both sets of data were small, reflected in the large degree of uncertainty in the estimates. For rotavirus and astrovirus, the Bayesian model gave somewhat lower estimates of hospital admissions, which might indicate that hospitalisations for these two pathogens are over-reported in outbreak data or that they were underascertained in the IID studies. Additionally, outbreaks might occur in specific age groups or individuals with underlying conditions or be due to high-dose exposure. Outbreak reports, however, contain limited information on the populations affected.

Using outbreak data to attribute cases of IID to foodborne transmission relies on certain assumptions, principally that outbreak cases reflect the epidemiology in the wider community. Another potential limitation is that there might be a bias towards investigation or reporting of foodborne outbreaks compared with outbreaks transmitted through other routes, like person-to-person transmission. This, however, does not seem to be the case: there has been a gradual decrease in the proportion of reported outbreaks involving foodborne transmission, which reflects both a reduction in incidence of certain foodborne pathogens, particularly *Salmonella*, and greater investigation of outbreaks in other settings, particularly viral outbreaks in hospitals and residential institutions.^18^ ^19^

Our study focused on foodborne illness burden in the general UK population. Some pathogens, however, are a particular problem among certain high-risk groups, such as *Listeria* among immunocompromised patients and pregnant women and rotavirus among children under 5 years. Our analysis was not designed to estimate burden in these subgroups, because our data sources contain limited information on these groups, and the size of some of these high-risk populations is uncertain. However, further studies to estimate burden in these groups is warranted.

We were unable to include other relevant pathogens such as toxoplasmosis, hepatitis A, hepatitis E and non-O157 VTEC in our analysis, due to a lack of relevant data in the UK. In a Dutch study *Toxoplasma gondii* caused the highest foodborne disease burden as measured by disability-adjusted life years, reflecting the importance of congenital toxoplasmosis.^16^

Our modelling approach meant we could use data from various sources to incorporate the best available information from the UK and elsewhere. Comparing models with and without prior information indicates where there is disagreement between data sources and enables uncertainty in all the relevant parameters to be accounted for. Uncertainty in these models reflects not simply statistical uncertainty in individual parameters, but disagreement between data sources and availability of information from previous studies. Information from previous studies on the proportion of IID transmitted through food was captured using Bayesian uniform priors. This is probably conservative, as it presupposes that every value within the specified limits is likely equal. For most pathogens, however, the number of available studies was small and using more informative priors was difficult to justify. The exception was *Campylobacter*, for which 14 studies contained relevant data. Even so, using data from risk factor studies presents problems in interpretation. Study design, methods and risk factors investigated varied widely. Consequently, variability between studies in the importance of food-related risk factors is high. The choice of Bayesian priors in estimation is necessarily a subjective process, as it depends on analysts’ confidence in the available information. Establishment of a process to develop greater international consensus on the choice of priors for individual pathogens could help to refine future estimates. Better baseline estimates would also inform predictions of the likely increase in foodborne disease due to climate change.^20^

We did not estimate deaths attributable to foodborne illness, due to the lack of reliable data sources on pathogen-specific mortality rates. Death certificates rarely provide information on specific gastrointestinal pathogens, while deaths in outbreaks are rare and may not be recorded if they occur sometime after the event. More generally, mortality estimates would be difficult to interpret. Deaths attributed to foodborne disease are not necessarily the same as preventable deaths. More focused epidemiological studies on mortality following IID would be helpful.

Our estimates measure foodborne disease burden only in the acute phase of illness. For some pathogens, the long-term consequences of illness can add considerably to their burden, for example *E. coli* O157-associated haemolytic uraemic syndrome and *Campylobacter*-associated Guillain-Barré syndrome.^21^ ^22^ Moreover, our estimates are based only on the number of cases of illness, and take no account of the consequences of illness in different sectors of the population. Further studies using additional measures of disease burden and taking into account long-term health consequences are therefore required.

Modelling is not necessarily a substitute for acquiring good quality primary data but it is very useful for pointing to important data gaps and major areas of uncertainty where primary data collection might be focused.

Controlling foodborne disease is an important policy issue. Given the burden of illness caused, there needs to be a continued focus on reducing illness due to *Campylobacter*, *Salmonella*, *C. perfringens* and norovirus.
