# Supplementary material for: Modelling study to estimate the health burden of foodborne diseases: cases, general practice consultations and hospitalisations in the UK, 2009
Source: BMJ Open. 2016 Sep 13;6(9):e011119. doi: 10.1136/bmjopen-2016-011119 (PMC5030535; doi:10.1136/bmjopen-2016-011119)
Supplement: Supplementary technical appendix [file bmjopen-2016-011119supp_appendix.pdf]

Technical appendix

Bootstrapping of outbreak data to estimate the proportion of cases due to foodborne transmission

For each pathogen, we estimated the proportion of cases attributable to foodborne transmission by bootstrapping 4,999 replicate samples from the outbreak dataset and obtaining an empirical distribution for the proportion of cases involved in foodborne outbreaks. For *Giardia* and *Cryptosporidium*, this gave unrealistically high values for the proportion attributable to foodborne transmission and we instead based our estimates on the proportion of outbreaks that were foodborne. We summarised the resulting distributions by fitting a Beta distribution using maximum likelihood.

For *Listeria*, for which the six cases observed were all from outbreaks involving foodborne transmission, we set a lower boundary for the proportion foodborne by assuming that the next outbreak observed, involving two cases, would not be foodborne. This is based on the definition for a general outbreak as an incident involving two or more epidemiologically-related cases. We then drew values at random from a Binomial distribution with 8 observations and 6 successes and fitted a Beta function to the resulting distribution. Similarly, for astrovirus, for which no foodborne outbreaks were reported, we set an upper boundary for the proportion foodborne by assuming that the next outbreak, involving two cases, would be foodborne, and derived Beta parameters in a similar fashion. The parameters from the fitted Beta distributions were then used in the Monte Carlo simulation. For adenovirus and sapovirus, for which no outbreaks were reported, we used Beta parameters derived from analysis of rotavirus and norovirus outbreaks respectively.

Bootstrapping of outbreak data to estimate the proportion of cases hospitalised

Data on hospitalisation in outbreaks were only available from England and Wales. For each reported outbreak in the England and Wales dataset (excluding outbreaks that occurred in hospitals and residential institutions), we calculated the proportion of outbreak cases that was hospitalised and plotted the resulting distribution for the proportion of cases hospitalised. We calculated this by causative organism and separately for all outbreaks and for foodborne outbreaks only. There was no major difference in hospitalisation between all outbreaks and foodborne outbreaks, so we based estimates of hospitalisation on data from all outbreaks. To account for uncertainty in hospitalisation parameters, we used a two-step approach. For each pathogen, we first obtained an empirical distribution for the proportion of cases hospitalised by bootstrapping 4,999 replicate samples of the outbreak data. For example, if there were 50 reported outbreaks for a given pathogen, we sampled 50 outbreaks with replacement from this set and calculated the mean proportion of cases hospitalised across the outbreak sample, weighted by outbreak size. This was repeated 4,999 times for each pathogen. The hospitalisation proportion was weighted by outbreak size because many reported outbreaks involve few cases and are therefore unlikely to involve hospitalised cases. The small number of larger outbreaks, on the other hand, is potentially more informative for estimating hospitalisation. We then fitted a Beta distribution to the bootstrapped data and estimated the corresponding a and b parameters using maximum likelihood. The mean hospitalisation proportions for each pathogen and Beta parameters used in Model 1 are given in Table A1. The fits of the Beta distributions to the outbreak data are show graphically in Figure A1.

For *Listeria*, all reported outbreaks occurred in hospitals, so it was not possible to estimate the hospitalisation rate from outbreaks. For adenovirus and sapovirus, no outbreaks were reported. For these two pathogens, parameters based on analysis of rotavirus and norovirus outbreaks respectively were used. Bootstrap estimates with fitted Beta distributions for the remaining 10 pathogens are shown below.

Figure A1: Bootstrap estimates of the proportion of cases hospitalised with fitted Beta distributions (shown by red line) by pathogen, UK outbreak data 2001-08

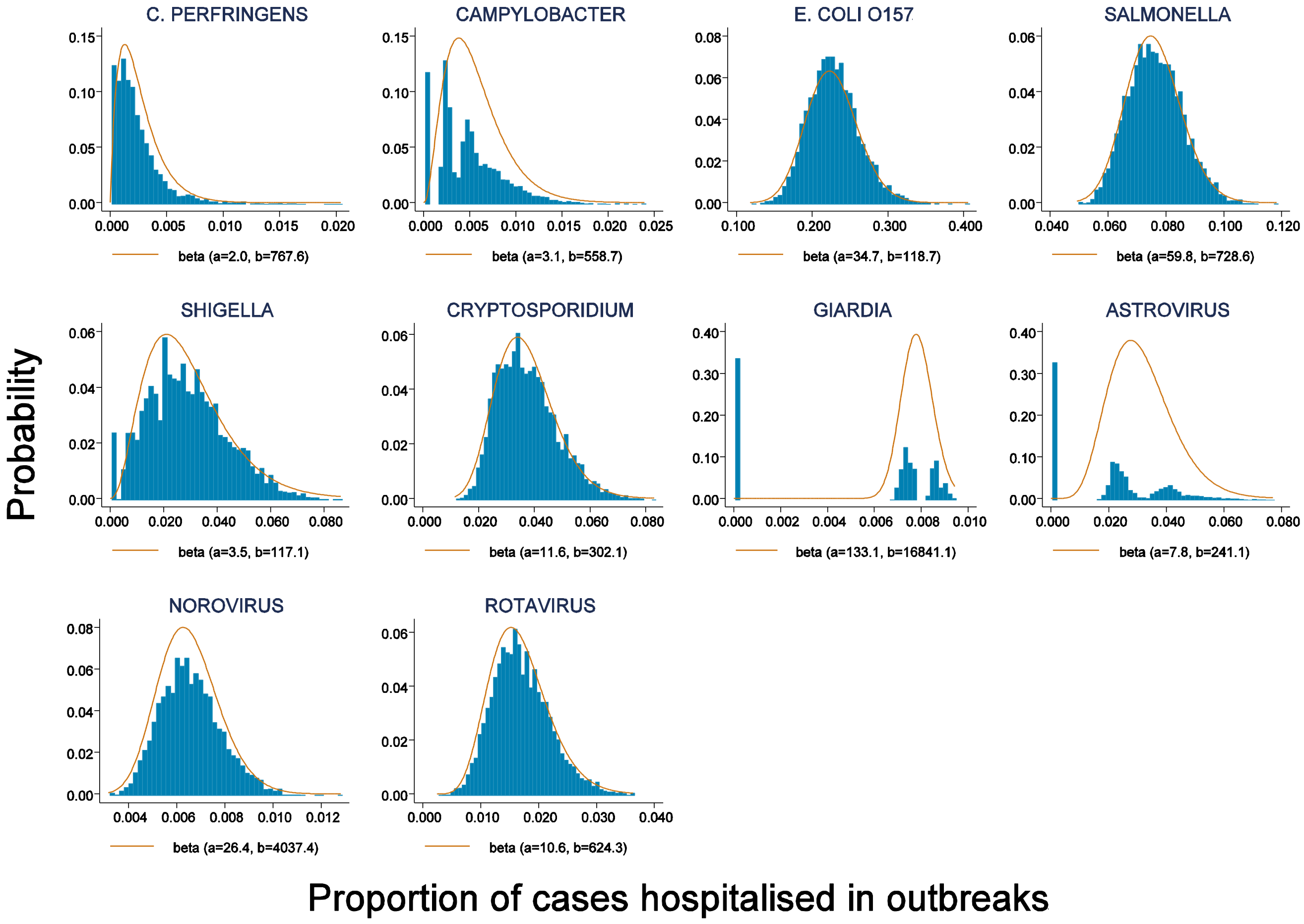

Deriving priors for the proportion hospitalised (γ_p) from the IID1 and IID2 Studies

We pooled data from the IID1 and IID2 Studies and calculated, by pathogen, the proportion of cases presenting to the GP that were hospitalised. Applying this proportion to the rate of GP consultation gave an estimate of the hospitalisation rate. For each pathogen, we used the ratio of this rate to the rate of community IID to obtain an estimate of the proportion of cases hospitalised. This approach implicitly assumes that hospitalised cases always consult a GP. This is reasonable in the UK, as hospitalisation is likely to occur through a GP referral, but potentially disregards a fraction of more severe cases (e.g. cases admitted as a result of an emergency hospital visit). However, it was not possible to estimate hospitalisation directly from the IID1 and IID2 community cohort study components, as hospitalisation is very uncommon and the two cohort studies were not designed to measure the rate of hospitalisation.

To account for uncertainty in the hospitalised proportion, we took 100,000 random samples from the distributions of the overall IID rate, c_p, and the proportion of GP cases hospitalised, and fitted a Beta function to the resulting distribution for the hospitalised proportion using maximum likelihood methods. The estimated parameters from this Beta distribution were used to inform the prior values for γ_p in the Bayesian approach (Table A2). For VTEC O157, for which hospitalisation information was not available from IID1 and IID2, we used a non-informative prior defined by the distribution Beta(1,1). For pathogens for which no hospitalisations were observed in the IID1 and IID2 studies, we specified limits to the fitted Beta distributions by assuming that the next case observed would have been hospitalised. Thus, for *Shigella*, with 11 cases and no hospitalisations, we obtained Beta parameters for a distribution with a mean equivalent to 1/12=0.087. Empirical bootstrap distributions with fitted Beta functions are shown below.

Monte Carlo approach (Model 1)

We obtained estimates of F_p, G_p and H_p using Monte Carlo simulation, each time drawing at random from each parameter distribution in the model. We carried out 100,000 simulations, discarding the first 10% and retaining the model outputs for every 10th simulation. We checked model convergence graphically by plotting parameter values over time to verify adequate mixing, plotting autocorrelograms and comparing density plots for outcome variables by tertile of the simulation chain. The model and associated parameter distributions are described below:

$$F_p = N \cdot c_p \cdot \pi_p$$
$$G_p = N \cdot g_p \cdot \pi_p$$
$$H_p = F_p \cdot \gamma_p$$

$$\log(c_p) \sim N(\mu_{cp}, \sigma_{cp})$$

$$\log(g_p) \sim N(\mu_{gp}, \sigma_{gp})$$

$$\pi_p \sim \text{Beta}(a_{\pi p}, b_{\pi p})$$

$$\gamma_p \sim \text{Beta}(a_{\gamma p}, b_{\gamma p})$$

From the ensuing distributions of F_p, G_p and H_p, we used the median and central 95% of the distributions as the point estimates and 95% credible intervals respectively. Parameter values for each pathogen are given in table A1 below.

Bayesian approach (Models 2 and 3)

In the Bayesian approach, we included parameters for the prior distributions of π_p and γ_p. These priors were used, together with the outbreak data to obtain posterior distributions for these parameters, which were then used in the model as described below:

$$F_p = N \cdot c_p \cdot \pi_p$$
$$G_p = N \cdot g_p \cdot \pi_p$$
$$H_p = F_p \cdot \gamma_p$$

$$\log(c_p) \sim N(\mu_{cp}, \sigma_{cp})$$

$$\log(g_p) \sim N(\mu_{gp}, \sigma_{gp})$$

$$f_p \sim \text{Binomial}(\pi_p, o_p)$$

$$\pi_p \sim \text{uniform}(u_{\pi p}, v_{\pi p})$$

$$h_p \sim \text{Binomial}(\gamma_p, m_p)$$

$$\gamma_p \sim \text{Beta}(a_{\gamma p}, b_{\gamma p})$$

For each pathogen, p, the parameters f_p and o_p represent the number of cases involved in foodborne and all outbreaks respectively. Similarly, h_p and m_p represent the pathogen-specific number of hospitalisations and GP consultations as observed in IID1 and IID2. The prior values for parameters π_p and γ_p are defined by uniform and Beta distributions respectively as described above. In Model 2, the uniform distributions for π_p were informed by data from published multi-pathogen food attribution studies. We used a further model, Model 3, with the same structure as Model 2, but with parameters for the prior distribution of π_p being derived from case-control and food attribution studies from the literature review. A full description of parameters for models 2 and 3 is given in the technical appendix.

For each model, we carried out 100,000 simulations to obtain posterior distributions for F_p, G_p and H_p, discarding the first 10% and retaining the model outputs for every 10th simulation. We checked for model convergence as described for the Monte Carlo approach above. Parameter values for each pathogen are given in tables A2 and A3 below.

Table A1: Parameter values for Model 1

	Incidence					Proportion foodborne				Proportion hospitalised			
Organism	μ_{cp}	σ_{cp}	μ_{gp}	σ_{gp}	Source	PF	$a_{\pi p}$	$b_{\pi p}$	Source	PH	$a_{\gamma p}$	$b_{\gamma p}$	Source
Bacteria													
C. perfringens	-6.50	0.49	-8.34	0.39	A	0.862	25.0	4.3	D	0.0017	2.0	767.6	D
Campylobacter	-4.68	0.22	-6.66	0.18	A	0.501	6.8	6.5	D	0.0046	3.1	558.7	D
E. coli O157	-8.11	1.36	-11.51	1.12	A	0.531	14.1	12.8	D	0.2235	34.7	118.7	D
Listeria	--	--	--	--	C	1.000	7.8	3.1	D	--	--	--	H
Salmonella	-7.42	0.71	-8.62	0.46	A	0.904	116.0	12.6	D	0.0751	59.8	728.6	D
Shigella	-9.29	0.97	-9.98	0.27	B	0.222	1.7	4.7	D	0.0260	3.5	117.1	D
Protozoa													
Cryptosporidium	-7.26	0.69	-8.52	0.45	A	0.051	4.0	73.2	D	0.0362	11.6	302.1	D
Giardia	-7.13	0.67	-9.32	0.56	A	0.167	4.0	11.8	D	0.0073	133.1	16,841.1	D
Viruses													
Adenovirus	-4.59	0.21	-7.08	0.28	A	--	4.8	230.3	F	--	10.6	624.3	F
Astrovirus	-5.24	0.29	-7.82	0.37	A	0.000	3.6	437.6	D	0.2222	7.8	241.1	D
Norovirus	-3.06	0.09	-6.18	0.19	A	0.025	38.7	1,473.6	D	0.0064	26.4	4,037.4	D
Rotavirus	-4.37	0.19	-6.60	0.21	A	0.014	4.8	230.3	D	0.0165	10.6	624.3	D
Sapovirus	-3.65	0.13	-6.46	0.19	A	--	38.7	1,473.6	G	--	26.4	4,037.4	G

PF: Proportion foodborne as estimated from outbreak data; PH: Proportion hospitalised as estimated from outbreak data

A: IID2 Study; B: 2009 laboratory reports * IID1 reporting ratio; C: 2009 laboratory reports

D: Outbreak data; F: No outbreak data available, assumed same as rotavirus; G: No outbreak data available, assumed same as norovirus

H: All reported Listeria outbreaks were in hospitals/residential institutions so hospitalisation parameters could not be estimated

Table A2: Parameter values for Model 2

	Proportion foodborne						Proportion hospitalised					
	Binomial likelihood			Uniform prior			Binomial likelihood			Beta prior		
Organism	f_p	o_p	Source	$u_{\pi p}$	$v_{\pi p}$	Source	h_p	m_p	Source	$a_{\gamma p}$	$b_{\gamma p}$	Source
Bacteria												
C. perfringens	1,691	1,964	D	0.761	1.000	[1–7]	2	1,120	D	1.6	277.1	J
Campylobacter	373	761	D	0.420	0.800	[1–7]	2	424	D	3.5	2,119.3	J
E. coli O157	564	1,041	D	0.400	0.760	[1–7]	197	877	D	1.0	1.0	K
Listeria	6	8	D	0.690	1.000	[1,4–6,8]	--	--	H	--	--	
Salmonella	7,128	7,892	D	0.550	0.950	[1–7]	419	5,527	D	1.2	75.3	J
Shigella	65	310	D	0.082	0.310	[1,5,6,9]	4	153	D	0.9	7.1	J
Protozoa												
Cryptosporidium	4	65	D	0.000	0.120	[1–3,5,9]	31	836	D	1.2	99.1	J
Giardia	1	7	D	0.050	0.300	[1–3,5,7]	1	137	D	1.2	150.4	J
Viruses												
Adenovirus	30	2,338	F	0.000	0.100	[1,2]	20	1,211	F	3.1	1,819.8	J
Astrovirus	2	285	D	0.005	0.107	[1,2,5]	2	88	D	2.5	1,252.6	J
Norovirus	1500	58,855	D	0.000	0.390	[1–3,5–7,9,10]	80	12,333	D	3.2	6,124.2	J
Rotavirus	30	2,338	D	0.005	0.130	[1–3,5,7]	20	1,211	D	3.6	1,295.6	J
Sapovirus[1]	1500	58,855	G	--	--		80	12,333	G	3.9	3,072.6	J

Incidence parameters are the same as those for Model 1

[1] Estimates for sapovirus could not be calculated from this model because of the lack of published data to inform prior parameters

D: Outbreak data; F: No outbreak data available, assumed same as rotavirus; G: No outbreak data available, assumed same as norovirus

H: All reported Listeria outbreaks were in hospitals/residential institutions so hospitalisation parameters could not be estimated

J: IID1 and IID2 GP Presentation Studies; K: Non-informative Beta distribution used

Table A3: Parameter values for Model 3

	Proportion foodborne						Proportion hospitalised					
	Binomial likelihood			Uniform prior			Binomial likelihood			Beta prior		
Organism	f_p	o_p	Source	$u_{\pi p}$	$v_{\pi p}$	Source	h_p	m_p	Source	$a_{\gamma p}$	$b_{\gamma p}$	Source
Campylobacter	373	761	D	0.110	1.000	[11–26]	2	424	D	3.5	2,119.3	J
E. coli O157	564	1,041	D	0.090	0.642	[13,27–29]	197	877	D	1.0	1.0	K
Listeria	6	8	D	0.180	1.000	[30,31]	--	--	H	1.0	1.0	K
Salmonella	7,128	7,892	D	0.090	1.000	[13,32–34]	419	5,527	D	1.2	75.3	J

Incidence parameters are the same as those for Model 1

D: Outbreak data

H: All reported Listeria outbreaks were in hospitals/residential institutions so hospitalisation parameters could not be estimated

J: IID1 and IID2 GP Presentation Studies; K: Non-informative Beta distribution used

References to papers included in the systematic review

1 Adak GK, Long SM, O'Brien SJ. Trends in indigenous foodborne disease and deaths, England and Wales: 1992 to 2000. *Gut* 2002;**51**:832–41. doi:10.1136/gut.51.6.832

2 Hall G, Kirk MD, Becker N, *et al.* Estimating foodborne gastroenteritis, Australia. *Emerg Infect Dis* 2005;**11**:1257–64. doi:10.3201/eid1108.041367

3 Havelaar AH, Galindo AV, Kurowicka D, *et al.* Attribution of foodborne pathogens using structured expert elicitation. *Foodborne Pathog Dis* 2008;**5**:649–59. doi:10.1089/fpd.2008.0115

4 Lake RJ, Cressey PJ, Campbell DM, *et al.* Risk ranking for foodborne microbial hazards in New Zealand: burden of disease estimates. *Risk Anal* 2010;**30**:743–52. doi:10.1111/j.1539-6924.2009.01269.x

5 Scallan E. Foodborne Illness Acquired in the United States—Major Pathogens. *Emerg Infect Dis* 2011;**17**:1–38. doi:10.3201/eid1701.P11101

6 Vaillant V, de Valk H, Baron E, *et al.* Foodborne infections in France. *Foodborne Pathog Dis* 2005;**2**:221–32. doi:10.1089/fpd.2005.2.221

7 Van Duynhoven Y, De Wit M, Kortbeek L, *et al.* Voedselinfecties in Nederland. *Ned Tijdschr Med Microbiol* 2002;**10**.

8 Ravel A, Davidson VJ, Ruzante JM, *et al.* Foodborne proportion of gastrointestinal illness: estimates from a Canadian expert elicitation survey. *Foodborne Pathog Dis* 2010;**7**:1463–72. doi:10.1089/fpd.2010.0582

9 Carrique-Mas J, Andersson Y, Hjertqvist M, *et al.* Risk factors for domestic sporadic campylobacteriosis among young children in Sweden. *Scand J Infect Dis* 2005;**37**:101–10. doi:10.1080/00365540510027165

10 Danis K, Renzi M Di, Neill WO, *et al.* Risk factors for sporadic Campylobacter infection: An all-Ireland case-control study. *Euro Surveill* 2009;**14**:1–8.

11 Denno DM, Keene WE, Hutter CM, *et al.* Tri-county comprehensive assessment of risk factors for sporadic reportable bacterial enteric infection in children. *J Infect Dis* 2009;**199**:467–76. doi:10.1086/596555

12 Doorduyn Y, Van Den Brandhof WE, Van Duynhoven Y, *et al.* Risk factors for indigenous Campylobacter jejuni and Campylobacter coli infections in The Netherlands: a case-control study. *Epidemiol Infect* 2010;**138**:1391–404. doi:10.1017/S095026881000052X

13 Effler P, Ieong MC, Kimura a, *et al.* Sporadic Campylobacter jejuni infections in Hawaii: associations with prior antibiotic use and commercially prepared chicken. *J Infect Dis* 2001;**183**:1152–5. doi:10.1086/319292

14 Evans MR, Ribeiro CD, Salmon RL. Hazards of healthy living: bottled water and salad vegetables as risk factors for Campylobacter infection. *Emerg Infect Dis* 2003;**9**:1219–25.http://www.pubmedcentral.nih.gov/articlerender.fcgi?artid=3033096&tool=pmcentrez&rendertype=abstract

15 Fajó-Pascual M, Godoy P, Ferrero-Cáncer M, *et al.* Case-control study of risk factors for sporadic Campylobacter infections in northeastern Spain. *Eur J Public Health* 2010;**20**:443–8.

doi:10.1093/eurpub/ckp206

16 Friedman CR, Hoekstra RM, Samuel M, *et al.* Risk factors for sporadic Campylobacter infection in the United States: A case-control study in FoodNet sites. *Clin Infect Dis* 2004;**38 Suppl 3**:S285–96. doi:10.1086/381598

17 Neimann J, Engberg J, Mølbak K, *et al.* A case-control study of risk factors for sporadic campylobacter infections in Denmark. *Epidemiol Infect* 2003;**130**:353–66.http://www.pubmedcentral.nih.gov/articlerender.fcgi?artid=2869971&tool=pmcentrez&rendertype=abstract

18 Rodrigues LC, Cowden JM, Wheeler JG, *et al.* The study of infectious intestinal disease in England: risk factors for cases of infectious intestinal disease with Campylobacter jejuni infection. *Epidemiol Infect* 2001;**127**:185–93.http://www.pubmedcentral.nih.gov/articlerender.fcgi?artid=2869737&tool=pmcentrez&rendertype=abstract

19 Sheppard SK, Dallas JF, Strachan NJC, *et al.* Campylobacter genotyping to determine the source of human infection. *Clin Infect Dis* 2009;**48**:1072–8. doi:10.1086/597402

20 Stafford RJ, Schluter PJ, Wilson AJ, *et al.* Population-Attributable Risk Estimates for Risk Factors Associated with Campylobacter. *Emerg Infect Dis* 2008;**14**:895–901.

21 Tam CC, Higgins CD, Neal KR, *et al.* Chicken consumption and use of acid-suppressing medications as risk factors for Campylobacter enteritis, England. *Emerg Infect Dis* 2009;**15**:1402–8. doi:10.3201/eid1509.080773

22 Unicomb LE, Dalton CB, Gilbert GL, *et al.* Age-specific risk factors for sporadic Campylobacter infection in regional Australia. *Foodborne Pathog Dis* 2008;**5**:79–85. doi:10.1089/fpd.2007.0047

23 Michaud S, Ménard S, Arbeit RD. Campylobacteriosis, Eastern Townships, Québec. *October* 2004;**10**:1844–7.

24 Wingstrand A, Neimann J, Engberg J, *et al.* Fresh Chicken as Main Risk Factor for Campylobacteriosis, Denmark. *Emerg Infect Dis* 2006;**12**:280–4.

25 Kassenborg HD, Hedberg CW, Hoekstra M, *et al.* Farm visits and undercooked hamburgers as major risk factors for sporadic Escherichia coli O157:H7 infection: data from a case-control study in 5 FoodNet sites. *Clin Infect Dis* 2004;**38 Suppl 3**:S271–8. doi:10.1086/381596

26 Voetsch a C, Kennedy MH, Keene WE, *et al.* Risk factors for sporadic Shiga toxin-producing Escherichia coli O157 infections in FoodNet sites, 1999-2000. *Epidemiol Infect* 2007;**135**:993–1000. doi:10.1017/S0950268806007564

27 Werber D, Behnke SC, Fruth A, *et al.* Shiga toxin-producing Escherichia coli infection in Germany: different risk factors for different age groups. *Am J Epidemiol* 2007;**165**:425–34. doi:10.1093/aje/kwk023

28 Little CL, Pires SM, Gillespie IA, *et al.* Attribution of Human Listeria monocytogenes Infections in England and Wales to Ready-to-Eat Food Sources Placed on the Market : Adaptation of the Hald. *Foodborne Pathog Dis* 2010;**7**.

29 Varma JK, Samuel MC, Marcus R, *et al.* Listeria monocytogenes infection from foods prepared in a commercial establishment: a case-control study of potential sources of sporadic illness in

the United States. *Clin Infect Dis* 2007;**44**:521–8. doi:10.1086/509920

30 Doorduyn Y, Van Den Brandhof WE, Van Duynhoven Y, *et al.* Risk factors for Salmonella Enteritidis and Typhimurium (DT104 and non-DT104) infections in The Netherlands: predominant roles for raw eggs in Enteritidis and sandboxes in Typhimurium infections. *Epidemiol Infect* 2006;**134**:617–26. doi:10.1017/S0950268805005406

31 Hald T, Vose D, Wegener HC, *et al.* A Bayesian approach to quantify the contribution of animal-food sources to human salmonellosis. *Risk Anal* 2004;**24**:255–69. doi:10.1111/j.0272-4332.2004.00427.x

32 Kimura AC, Reddy V, Marcus R, *et al.* Chicken consumption is a newly identified risk factor for sporadic Salmonella enterica serotype Enteritidis infections in the United States: a case-control study in FoodNet sites. *Clin Infect Dis* 2004;**38 Suppl 3**:S244–52. doi:10.1086/381576
